# Supplementary material for: Comparative gene expression analysis of Beauveria bassiana against Spodoptera frugiperda
Source: PeerJ. 2025 Jun 30;13:e19591. doi: 10.7717/peerj.19591 (PMC12225636; doi:10.7717/peerj.19591)
Supplement: Supplemental Information 1 [file peerj-13-19591-s001.docx]

**Table S1: List of primers used for RT-qPCR validation of differentially expressed genes**

| symbol | Annotation | Forward primer (5′-3′) | Reverse primer (5′-3′) |
| --- | --- | --- | --- |
| MFS2 | Siderophore iron transporter mirB | TCATGGACCGCACAGTCTTC | CCAGGTCACTTGATCGGCTT |
| STL1 | Sugar transporter STL1 | CTTCATTCTCGCGTTCGTGC | AGCGATGGCTGCGATAACAA |
| chi2 | Chitinase III | CGCAAAACGTCGTGTACTGG | GGGGATGTTGTTGTTGTCGC |
| CTF1-BETA | cutinase transcription factor | TAACACCGTCAACCCCAACC | CGTCAAGGCACCCTTCATCT |
| SLD | Fatty acid desaturase | CCCTCCTTGGCTGGACTTTG | CTTCTGCGCCTTTCGGAGAT |
| oca3 | tetratricopeptide-like protein | AAGCTGGCAGAGATTGTGCG | ACTTGGAAAGGAGCTCTCGC |
| K0381 | Catalase | CGTCTGCCCCATGATGAACT | CGAGACGTGCTCCTTGAACT |
| MGG-080 | metalloprotease-like protein | CGAGTTTTGCAAGACGGAGC | TTCGCCCTTGGCAATCTTGT |
| RV15333 | 2-nitropropane dioxygenase | GCGGTCTCGGTGTTATTGGT | AAAGGAGCCTTCTTGTCGTCG |
| SPCF3.16 | putative RING finger protein | CCAGTCCCCATCCCTACAGA | CGCAGCCATGATAGTAGGCA |
| FUM15 | cytochrome P450 3A9 | ATCGGTTTCAACCAGGCCAT | TTCCTCGACGTACTTGTGGC |
| rio2 | serine/threonine-protein kinase | GCAAGCTTTACGCGAGGAAG | TATAGCGCTGCAGGATTGGG |
| LIP | lipase | CACTATCGGCTTCGACCTCC | ACTTCTGGTCCTGCTTGCTC |
| K13524 | Amino butyrate aminotransferase | CTGTTTGGCTCCCTGTCCAC | GGTACTTGAGCTGCGGGAAG |
| Arc2 | β-actin | GCCTGATGGGCAAGTCAT | TGGGAGCAAGAGCAGTG |
