# Supplementary figures and images for: Comparative gene expression analysis of Beauveria bassiana against Spodoptera frugiperda
Source: PeerJ. 2025 Jun 30;13:e19591. doi: 10.7717/peerj.19591 (PMC12225636; doi:10.7717/peerj.19591)

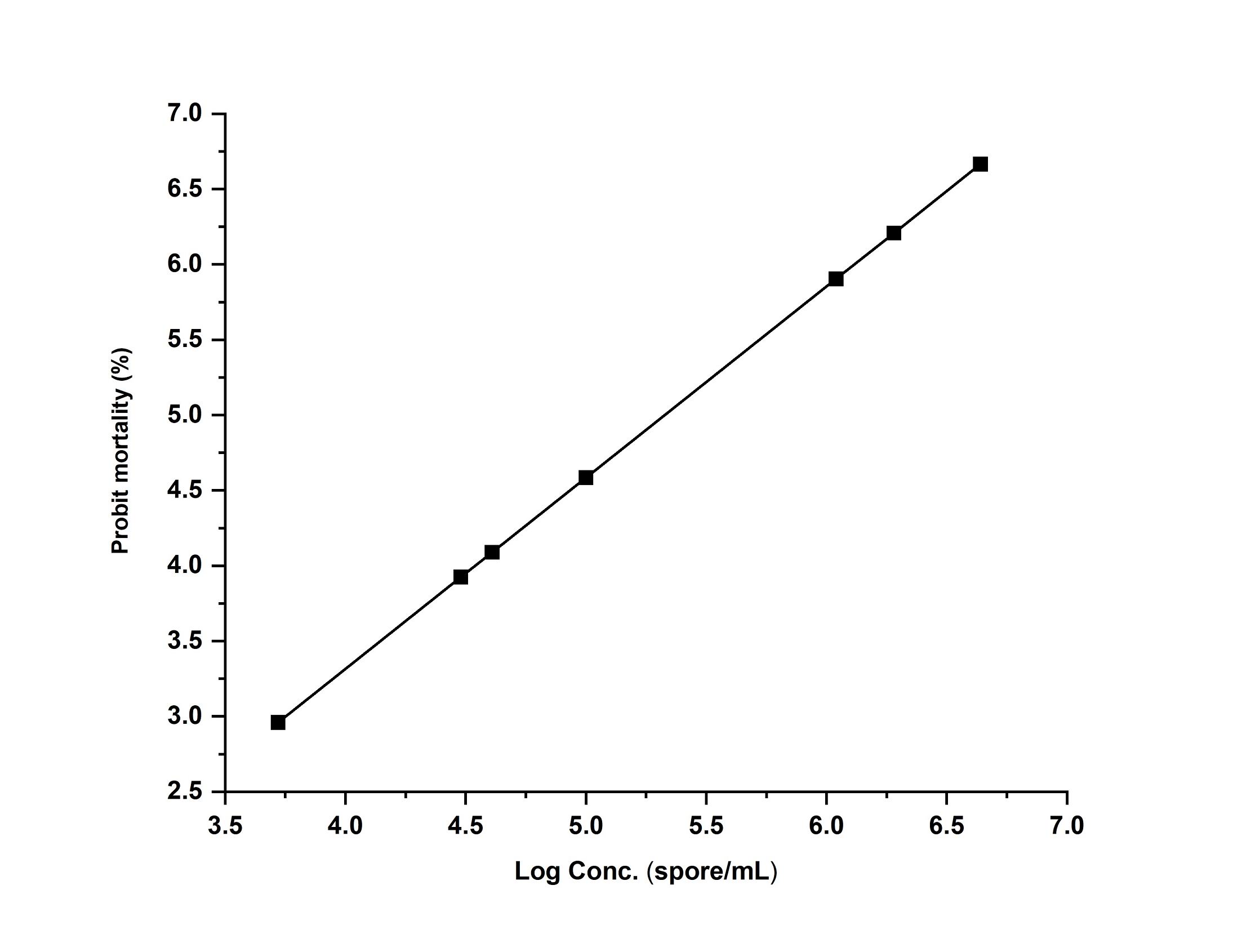

Supplement: Supplemental Information 2 [file peerj-13-19591-s002.jpg]
