# Supplementary material for: Comparative gene expression analysis of Beauveria bassiana against Spodoptera frugiperda
Source: PeerJ. 2025 Jun 30;13:e19591. doi: 10.7717/peerj.19591 (PMC12225636; doi:10.7717/peerj.19591)
Supplement: Supplemental Information 7 [file peerj-13-19591-s007.docx]

**Supporting Information for**

**Comparative gene expression analysis of *Beauveria bassiana* with or without host insect**

**Introduction**

The supplementary information below includes the primers that were designed for RT-qPCR in this experiment. These primers are listed in Table S1. Additionally, the estimated LC_50_ value for mortality in *S. frugiperda* is shown in Fig. S1.

**Table S1** The primer used in our study

| symbol | Annotation | Forward primer (5′-3′) | Reverse primer (5′-3′) |
| --- | --- | --- | --- |
| MFS2 | Siderophore iron transporter mirB | TCATGGACCGCACAGTCTTC | CCAGGTCACTTGATCGGCTT |
| STL1 | Sugar transporter STL1 | CTTCATTCTCGCGTTCGTGC | AGCGATGGCTGCGATAACAA |
| chi2 | Chitinase III | CGCAAAACGTCGTGTACTGG | GGGGATGTTGTTGTTGTCGC |
| CTF1-BETA | cutinase transcription factor | TAACACCGTCAACCCCAACC | CGTCAAGGCACCCTTCATCT |
| SLD | Fatty acid desaturase | CCCTCCTTGGCTGGACTTTG | CTTCTGCGCCTTTCGGAGAT |
| oca3 | tetratricopeptide-like protein | AAGCTGGCAGAGATTGTGCG | ACTTGGAAAGGAGCTCTCGC |
| K0381 | Catalase | CGTCTGCCCCATGATGAACT | CGAGACGTGCTCCTTGAACT |
| MGG-080 | metalloprotease-like protein | CGAGTTTTGCAAGACGGAGC | TTCGCCCTTGGCAATCTTGT |
| RV15333 | 2-nitropropane dioxygenase | GCGGTCTCGGTGTTATTGGT | AAAGGAGCCTTCTTGTCGTCG |
| K13524 | Amino butyrate aminotransferase | CTGTTTGGCTCCCTGTCCAC | GGTACTTGAGCTGCGGGAAG |
| Arc2 | β-actin | GCCTGATGGGCAAGTCAT | TGGGAGCAAGAGCAGTG |


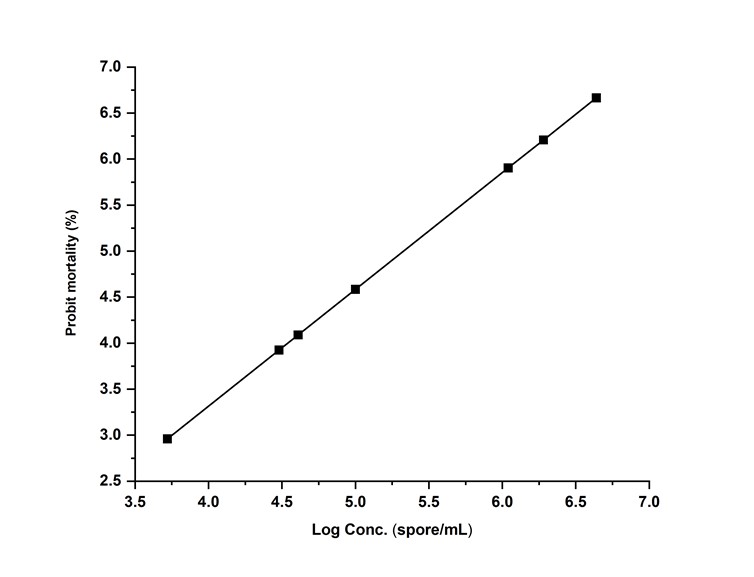


**Fig. S1.** Toxicity profile of *B. bassiana* CDL1 against FAW treated in the third larval instar.
